# Metabolomics in cardiometabolic diseases: Key biomarkers and therapeutic implications for insulin resistance and diabetes

**DOI:** 10.1111/joim.20090

**Published:** 2025-04-27

**Authors:** David Rizo‐Roca, John D. Henderson, Juleen R. Zierath

**Affiliations:** ^1^ Department of Physiology and Pharmacology, Integrative Physiology Karolinska Institutet Stockholm Sweden; ^2^ Novo Nordisk Foundation Center for Basic Metabolic Research Faculty of Health and Medical Sciences University of Copenhagen Copenhagen Denmark; ^3^ Department of Molecular Medicine and Surgery, Integrative Physiology Karolinska Institutet Stockholm Sweden

**Keywords:** biomarkers, cardiometabolic diseases, insulin resistance, metabolomics, therapeutic targets

## Abstract

Cardiometabolic diseases—including Type 2 diabetes and obesity—remain leading causes of global mortality. Recent advancements in metabolomics have facilitated the identification of metabolites that are integral to the development of insulin resistance, a characteristic feature of cardiometabolic disease. Key metabolites, such as branched‐chain amino acids (BCAAs), ceramides, glycine, and glutamine, have emerged as valuable biomarkers for early diagnosis, risk stratification, and potential therapeutic targets. Elevated BCAAs and ceramides are strongly associated with insulin resistance and Type 2 diabetes, whereas glycine exhibits an inverse relationship with insulin resistance, making it a promising therapeutic target. Metabolites involved in energy stress, including ketone bodies, lactate, and nicotinamide adenine dinucleotide (NAD⁺), regulate insulin sensitivity and metabolic health, with ketogenic diets and NAD⁺ precursor supplementation showing potential benefits. Additionally, the novel biomarker *N*‐lactoyl‐phenylalanine further underscores the complexity of metabolic regulation and its therapeutic potential. This review underscores the potential of metabolite‐based diagnostics and precision medicine, which could enhance efforts in the prevention, diagnosis, and treatment of cardiometabolic diseases, ultimately improving patient outcomes and quality of life.

## Graphical abstract

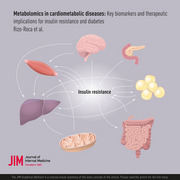

## Impact of cardiometabolic diseases and metabolite disruptions

Cardiometabolic diseases represent the leading cause of death worldwide, with ischemic heart disease alone representing 16% of global mortality [1]. Diabetes is the sixth cause of death from noncommunicable diseases [1] and is a prominent cardiovascular disease risk factor [2]. Although hyperglycemia remains the hallmark of diabetes and insulin resistance, the number of metabolites associated with Type 2 diabetes has increased over the past decade. Advances in metabolomics technologies have enabled the detection of increasingly lower concentrations of metabolites in progressively smaller biological samples. This has facilitated the identification of numerous metabolites and pathways disrupted in the context of insulin resistance, many of which serve as biomarkers and predictors of disease. By profiling the metabolic fingerprint of individuals, metabolomics allows for the detection of alterations that may precede the onset of clinical symptoms, rendering metabolites valuable tools for early diagnosis and risk stratification. Recent advances in metabolomics—a powerful tool for profiling metabolites in biological systems—have revolutionized our ability to detect early biomarkers and predict the onset of disease.

### Metabolites in insulin resistance: mechanisms and therapeutic implications

**Fig. 1:**
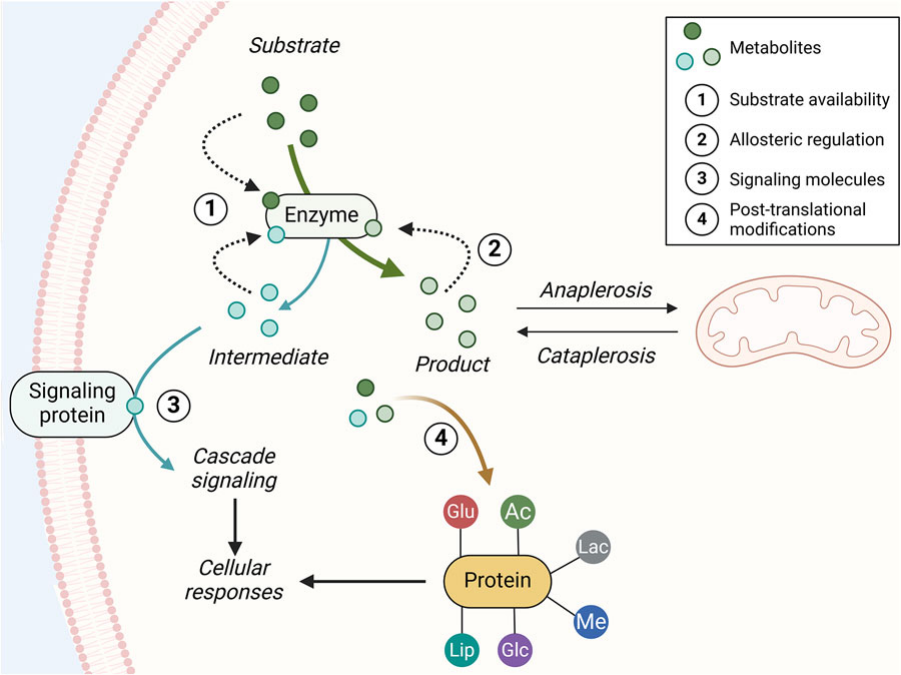
Metabolite‐mediated regulation of cellular function. Metabolites regulate cellular processes through four main mechanisms: (1) Substrate availability—metabolites act as precursors or intermediates in enzymatic reactions, thereby controlling the production of downstream products and influencing metabolic flux. (2) Allosteric regulation—metabolites bind to enzymes at non‐active sites, altering the conformation and activity of proteins to fine‐tune metabolic flux based on cellular demands. (3) Signaling molecules—metabolites act as signaling molecules by interacting with receptors or proteins to activate pathways that regulate cellular processes, such as energy balance, growth, and stress responses. (4) Post‐translational modifications—metabolites such as glucose (Glu), acetate (Ac), lipids (Lip), glucose derivatives (Glc), lactate (Lac), and methyl groups (Me) directly modify proteins, thereby altering function, stability, or localization, to regulate cellular activities. Additionally, the metabolic flux between anaplerosis (replenishment of intermediates) and cataplerosis (removal of intermediates) ensures mitochondrial metabolic balance. Therefore, alterations in metabolite levels can trigger widespread disruptions in cellular processes, amplifying effects through changes in enzyme activity, signaling pathways, and protein function. Source: Created in BioRender. Rizo Roca, D. (2025) https://BioRender.com/v67h675.

Historically, metabolites have been regarded as passive intermediates in metabolic pathways. However, these small molecules play a pleiotropic role in cell and systemic homeostasis. Metabolites act not only as substrates for metabolic pathways and protein post‐translational modifications but also as allosteric regulators of key enzymes involved in metabolic flux and as signaling molecules (Fig. 1). For example, alterations in lipid metabolites—such as ceramides and diacylglycerols—have been implicated in insulin resistance and the progression to Type 2 diabetes. These lipids disrupt insulin signaling pathways, impairing glucose uptake and utilization in skeletal muscle and adipose tissue. Similarly, disruptions in branched‐chain amino acid (BCAA) metabolism have been linked to insulin resistance through their effect on vascular fatty acid transport [3]. BCAAs have emerged as some of the most robust biomarkers for cardiometabolic diseases, including obesity and Type 2 diabetes [4]. Thus, in addition to their roles as biomarkers and disease predictors, metabolites contribute to the development or exacerbation of insulin resistance and other cardiometabolic diseases. Consequently, they represent therapeutic targets for the treatment or prevention of insulin resistance.

This review aims to examine the primary metabolite families associated with the development of Type 2 diabetes, current insights into the roles these metabolites play in molecular pathophysiology, and potential therapeutic interventions designed to modulate their activity (Table 1). The focus is on prospective studies that assess metabolite levels prior to the onset of disease. Altered metabolites detected before diabetes develops are more likely to be involved in disease pathophysiology, making these metabolites relevant for further investigation.

**Table 1:** Etiology and therapeutic prospects of metabolic alterations in Type 2 diabetes.

Metabolite	Etiological basis	Proposed pathogenic mechanism	Explored treatments
**Amino acids and derivatives**			
**↑** Branched‐chain amino acids	Impaired catabolism due to reduced enzyme activity (e.g., BCKDH)	Accumulation leads to mitochondrial dysfunction. Elevated 3‐HIB promotes fatty acid uptake and lipotoxicity in skeletal muscle	Dietary management and exercise improve BCAA metabolism. Activation of BCAA catabolism with sodium phenylbutyrate
**↑** Phenylalanine	Decreased liver phenylalanine hydroxylase activity	Modifies insulin receptor lysine residues, impairing signaling	A phenylalanine analog, phenylalaninol, improves glucose homeostasis in mice
**↓** Glutamine	Enhanced gluconeogenesis and glutamine utilization	Reduced glutamine impairs nitrogen balance, energy production, and mitochondrial function	Experimental use of glutamine supplementation
**↓** Glycine	High utilization in glutathione and methylation pathways	Low glycine levels reduce antioxidant capacity and metabolic flexibility, exacerbating insulin resistance	Glycine supplementation is under investigation
**↑** *N*‐Lactoyl‐phenylalanine	Lactate and amino acid conjugation are associated with metformin use	Levels reflect the use of metformin rather than directly driving pathogenesis	Exercise‐induced elevation is associated with health benefits
**Lipids**			
**↑** Sphingolipids (ceramides)	Increased de novo synthesis due to fatty acid overload and inflammation	Inhibits insulin signaling by impairing Akt phosphorylation; promotes lipotoxicity	Targeting enzymes controlling ceramide synthesis; dietary modifications
**↑** Acylcarnitines	Accumulation due to incomplete fatty acid oxidation	Reflects metabolic inefficiency, contributes to lipotoxicity and insulin resistance	Limited treatments: improving mitochondrial function via exercise or pharmacological agents
*Phosphatidylcholines (PCs)*			
**↓** One ester and one ether bonded	Depleted by oxidants	Reduced protection against oxidative stress and impaired mitochondrial function	Experimental use of antioxidant supplements
Dual‐ester bond	Increased VLDL synthesis due to elevated fatty acid availability	Contributes to lipotoxicity and insulin resistance through impaired lipid metabolism	Reducing dietary saturated fats
**↓** LysoPCs	Changes in lipid turnover due to insulin resistance and inflammation	Insulin secretagogue	
**↑** LysoalkylPCs		Altered interaction with G‐protein‐coupled receptors	
**Energy stress metabolites**			
**↑** Ketone bodies	Overproduction during lipolysis and energy stress	Serves as alternative energy sources; prolonged elevation can contribute to metabolic imbalance	Ketogenic diets are under investigation; tight glucose control
**↑** Lactate	Shift toward anaerobic glycolysis due to insulin resistance and hypoxia	Impairs glucose utilization; serves as a substrate for gluconeogenesis	Exercise to normalize lactate flux; no specific pharmacologic treatments available
**↓** NAD^+^	Impaired salvage pathway due to increased NNMT activity and metabolic stress	Reduced NAD^+^ availability impairs mitochondrial function, sirtuin activity, and redox homeostasis	NAD^+^ precursors (e.g., nicotinamide riboside) are under clinical evaluation

*Note*: Key metabolites implicated in Type 2 diabetes pathogenesis are summarized with their etiological basis, proposed pathogenic mechanisms, and potential therapeutic approaches. The table highlights dysregulations in amino acids, lipids, and energy stress metabolites, illustrating their roles in mitochondrial dysfunction, insulin resistance, and metabolic inefficiency, alongside explored treatments.

Abbreviations: BCKDH, branched‐chain α‐ketoacid dehydrogenase complex; 3‐HIB, 3‐hydroxyisobutyrate; VLDL, very low‐density lipoprotein; NAD^+^, nicotinamide adenine dinucleotide; NNMT, nicotinamide N‐methyltransferase

## Metabolite families implicated in Type 2 diabetes and insulin resistance

### Amino acids in insulin resistance: disruptions in metabolism and disease progression

**Fig. 2:**
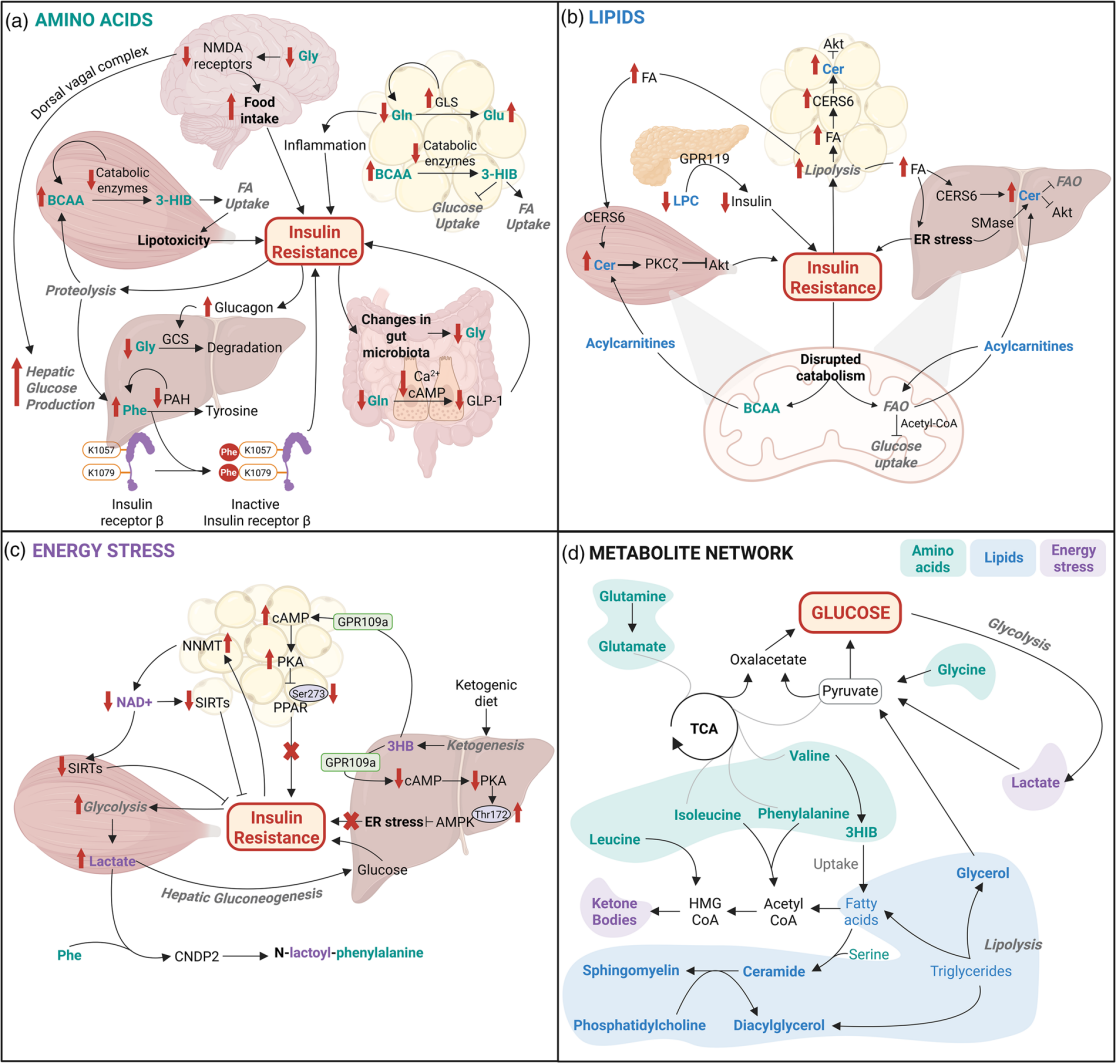
Metabolites associated with insulin resistance. (a) Amino acids—role of amino acids, particularly branched‐chain amino acids (BCAAs), glutamine (Gln), glycine (Gly), and phenylalanine (Phe), in insulin resistance. Impaired BCAA catabolism leads to the accumulation of intermediates such as 3‐hydroxyisobutyrate (3‐HIB), which facilitates fatty acid (FA) uptake and contributes to lipotoxicity in skeletal muscle. Low levels of glutamine are associated with inflammation and decreased production of glucagon‐like peptide‐1 (GLP‐1). Changes in gut microbiota and increased degradation lead to lower levels of glycine in individuals with insulin resistance. These changes are potentially associated with increased food intake and hepatic glucose production. Lysine phenylalanylation of the β‐subunit of the insulin receptor increases in a dose‐dependent manner and inhibits insulin signaling. (b) Lipids—Lipid metabolism and its impact on insulin resistance. Increased ceramide (Cer) synthesis contributes to insulin resistance through protein kinase C zeta (PKCζ)‐mediated inhibition of protein kinase B (Akt). Insulin resistance is further exacerbated by endoplasmic reticulum (ER) stress associated with lipid overload. Impaired BCAA catabolism and incomplete FA oxidation (FAO) enhance the production of acylcarnitines, leading to the concomitant exacerbation of Cer synthesis. Lysophosphatidylcholine (LPC) can activate G‐protein‐coupled receptor 119 (GPR119) and stimulate insulin secretion from pancreatic β‐cells. Therefore, lower circulating LPC levels observed in Type 2 diabetes may contribute to impaired insulin release and β‐cell dysfunction. (c) Energy stress—Perturbations in cellular energy homeostasis, including alterations in nicotinamide adenine dinucleotide (NAD^+^)‐dependent pathways and lactate production, may exacerbate insulin resistance. Increased reliance on glycolysis, especially under insulin‐resistant conditions, leads to enhanced lactate production, which in turn contributes to hepatic glucose production via the Cori cycle. Reduced NAD^+^ levels lead to decreased activity of insulin‐sensitizing sirtuins (SIRTs), such as SIRT1, impairing metabolic regulation and contributing to the development of insulin resistance. Under certain dietary conditions, such as caloric restriction or ketogenic diets, ketogenesis is enhanced, leading to the production of ketone bodies like β‐hydroxybutyrate (3HB), which can modulate key metabolic regulators such as peroxisome proliferator‐activated receptors (PPARs) and AMP‐activated protein kinase (AMPK), potentially improving insulin sensitivity and metabolic flexibility. (d) Metabolite network: Overview of the interconnected metabolite network associated with insulin resistance. This network illustrates the complex interplay between different metabolic pathways, including gluconeogenic and ketogenic amino acids, glycolysis, the tricarboxylic acid (TCA) cycle, ketogenesis, and lipolysis. CERS6, ceramide synthase 6; CNDP2, carnosine dipeptidase; FFA, free fatty acids; GCS, glycine cleavage system; GLS, glutaminase; Glu, glutamate; HMG‐CoA, 3‐hydroxy‐3‐methylglutaryl coenzyme A; NMDA, N‐methyl‐d‐aspartate; PKA, protein kinase A; PAH, phenylalanine hydroxylase; SMase, sphingomyelinase. Source: Created in BioRender. Rizo Roca, D. (2025) https://BioRender.com/v15d089.

Amino acids are increasingly recognized as key contributors to the development of insulin resistance and Type 2 diabetes. Disruptions in metabolic processes involving amino acid metabolism and protein turnover are evident in conditions such as insulin resistance, diabetes, and obesity, underscoring the role of metabolites in the pathophysiology of these disorders (Fig. 2a). Specifically, alterations in circulating BCAAs, aromatic amino acids, and glycine have been observed for more than five decades [5, 6]. Although amino acids in general play critical roles in insulin resistance, BCAAs stand out as key players, both as biomarkers and as contributors to metabolic dysfunction

### Branched‐chain amino acids: mediators of insulin resistance and therapeutic targets

Valine, leucine, and isoleucine are essential amino acids characterized by their branched side chains. Due to this structure, BCAAs undergo catabolism through a distinct pathway—primarily in skeletal muscle—in contrast to most other amino acids. Elevated circulating levels of BCAA have been associated with metabolic disorders, particularly insulin resistance and obesity [7, 8, 9, 10, 11]. Notably, plasma concentrations of all three BCAAs have been identified as predictive biomarkers of Type 2 diabetes, often years before diagnosis [12].

The elevated levels of BCAAs observed in conditions of glucose intolerance are likely attributable to reduced gene expression of key genes in BCAA catabolism in skeletal muscle [13]. A genome‐wide study has highlighted the importance of the *PPM1K* gene in this process [14]. *PPM1K* encodes a mitochondrial phosphatase that activates the branched‐chain alpha‐keto acid dehydrogenase complex, a rate‐limiting step in BCAA catabolism. Consequently, impaired skeletal muscle BCAA catabolism is associated with the development of Type 2 diabetes. Additionally, RNA sequencing of adipose biopsies from monozygotic twin pairs—one obese and one healthy—revealed a downregulation of BCAA catabolic genes in the obese twins [15], which was concomitant with higher plasma BCAA levels [16]. Furthermore, individuals who underwent Roux‐en‐Y gastric bypass surgery showed both a reduction in circulating BCAAs and an increase in the content of BCAA aminotransferase and branched‐chain alpha‐keto acid dehydrogenase in adipose biopsies [17]. This finding further suggests that adipose tissue, in conjunction with skeletal muscle, serves as an important site for BCAA catabolism. Beyond changes in the expression of BCAA catabolic enzymes, insulin resistance also leads to a disrupted proteolysis/protein synthesis balance that further contributes to elevated circulating BCAA levels [18].

The breakdown product of valine, 3‐hydroxyisobutyrate (3‐HIB), is elevated in skeletal muscle biopsies from patients with Type 2 diabetes [3]. Circulating levels of 3‐HIB correlate with hyperglycemia and insulin resistance and are reduced in obese patients following bariatric surgery [19]. Elevated 3‐HIB levels promote skeletal muscle fatty acid uptake, which subsequently increases diacylglycerol content and impairs Akt‐mediated insulin signaling [3]. Similarly, 3‐HIB enhances fatty acid uptake in both hepatocytes [20] and adipocytes [19], while impairing insulin‐stimulated glucose uptake in the adipocytes [19]. Conversely, reducing the expression of 3‐hydroxyisobutyryl‐CoA hydrolase improves insulin signaling in hepatocytes [20]. In addition to 3‐HIB, BCAA‐derived branched‐chain α‐keto acids also inhibit insulin‐stimulated glucose uptake in myotubes [21, 22]. Thus, elevated intracellular levels of BCAA intermediates may contribute to the development of insulin resistance.

Disentangling the precise effects of BCAAs on human metabolism is complicated by the challenges of administering controlled diets with varying BCAA content while maintaining both isocaloric and isonitrogenous conditions. Consequently, studies that employ such an approach are limited. Reducing BCAA content in an isocaloric diet lowers postprandial insulin secretion without affecting insulin sensitivity [23], whereas BCAA supplementation does not affect glucose uptake or insulin secretion in overweight individuals [24, 25]. In contrast, pharmacological activation of BCAA catabolism using sodium phenylbutyrate reduces plasma BCAA and glucose levels, improves peripheral insulin sensitivity, and enhances muscle mitochondrial oxidative capacity in individuals with Type 2 diabetes [26]. Although further clinical trials are needed, these findings suggest that targeting BCAA metabolism may be a promising strategy for treating insulin resistance.

### Aromatic amino acids: pathophysiological roles of phenylalanine in insulin resistance and glucose homeostasis

In addition to BCAAs, aromatic amino acids—particularly phenylalanine—have also been linked to insulin resistance, with mechanisms that may further unravel the pathophysiology of Type 2 diabetes. Phenylalanine is an essential amino acid and a precursor for tyrosine, dopamine, norepinephrine, and epinephrine. Along with BCAAs, phenylalanine was among the first amino acids to be linked with Type 2 diabetes [5]. The association between phenylalanine and the risk of Type 2 diabetes has been extensively validated over the past few decades through multiple cohorts and prospective studies [12, 27–29]. Furthermore, phenylalanine is not only linked to insulin resistance but also with reduced insulin secretion [30].

As with other amino acids, elevated plasma levels of phenylalanine may result from the dysregulation of protein turnover and amino acid catabolism in skeletal muscle and liver, conditions commonly observed in Type 2 diabetes. Additionally, a single nucleotide polymorphism in the phenylalanine hydroxylase gene (rs2245360, AA genotype) has been linked to an increased incidence of impaired fasting glucose [31]. Phenylalanine hydroxylase is the rate‐limiting enzyme in the liver that catalyzes the conversion of phenylalanine to tyrosine; thus, mutations in this gene may lead to the accumulation of phenylalanine in the blood. Conversely, increased phenylalanine hydroxylase expression and elevated phenylalanine hydroxylation rates have been observed in liver cells from streptozotocin‐induced diabetic rats [32]. These seemingly contradictory results may be attributed to the specific characteristics of streptozotocin‐induced models of diabetes or the possibility that compensatory increases in phenylalanine hydroxylation are insufficient to fully metabolize the excess phenylalanine.

Phenylalanine impairs insulin signaling and glucose uptake by modifying lysine residues 1057 and 1079 on the β subunit of the insulin receptor [33]. Lysine phenylalanylation, along with the inhibition of insulin signaling, increases in a phenylalanine dose‐dependent manner in human white blood cells. Consistently, elevated lysine phenylalanylation has been observed in white blood cells from individuals with Type 2 diabetes compared to matched controls, and this modification correlates with HbA1c. Furthermore, treatment of human hepatocytes with phenylalaninol, a phenylalanine analog, resulted in reduced lysine phenylalanylation and enhanced glucose uptake. Similarly, mice fed a phenylalaninol‐supplemented diet demonstrated improved insulin signaling, reduced body weight, and lower blood glucose levels. However, in healthy individuals, acute administration of phenylalanine suppresses energy intake and reduces plasma glucose in response to a meal challenge [34]. Thus, similar to BCAAs, the effects of amino acid supplementation can vary considerably depending on the metabolic health of the individual, as well as the dosage and duration of supplementation.

### Glycine: metabolic insights and potential for therapeutic intervention in Type 2 diabetes

Glycine is a non‐essential, proteinogenic, and glucogenic amino acid. In contrast to most biomarkers and predictors of Type 2 diabetes—which are typically elevated due to impairments in key enzymes within corresponding metabolic pathways—glycine levels exhibit an inverse association with insulin resistance [5]. Consequently, reduced plasma glycine concentrations have been consistently observed in individuals with pre‐diabetes and diabetes for decades [5, 35–37], and it serves as a strong predictor of incident Type 2 diabetes [35, 36]. In contrast, plasma glycine levels increase following exercise and bariatric surgery, correlating with improved insulin sensitivity [38, 39]. Notably, glycine levels are positively associated with insulin secretion in individuals with Type 2 diabetes [30], suggesting that this amino acid may play a role in the pathogenesis of insulin resistance.

Although substantial evidence indicates glycine levels are reduced in Type 2 diabetes, the underlying cause of this alteration remains unclear. Proposed mechanisms include alterations in gut microbiota in patients with Type 2 diabetes and the metabolism of glycine by bacteria [40], glucagon‐stimulated hepatic glycine degradation [41, 42], and increased urinary excretion of glycine precursors such as betaine [43]. Additionally, glycine utilization may be enhanced due to increased conjugation with fatty acids and BCAA catabolic intermediates, both of which are elevated in Type 2 diabetes. As glycine serves as an acceptor of acyl groups from these intermediates, this could contribute to a reduction in plasma glycine levels [44].

In individuals with Type 2 diabetes, glutathione synthesis and content are reduced up to 60% and 50%, respectively [45, 46]. Glutathione is the most abundant non‐enzymatic antioxidant and is synthesized from glycine, cysteine, and glutamate. Therefore, reduced glycine availability may limit glutathione synthesis. Consistent with this, dietary supplementation with glycine and cysteine partially restores glutathione synthesis in individuals with diabetes [45]. In animal models, glycine supplementation stimulates glutathione biosynthesis and mitigates atherosclerosis [47]. Thus, reduced plasma glycine levels may both reflect and exacerbate the increased oxidative stress characteristic of Type 2 diabetes. Furthermore, glycine enhances the activation of *N*‐methyl‐d‐aspartate receptors in the brainstem dorsal vagal complex, inhibiting hepatic glucose production [48] and reducing food intake [49]. Glycine is also a substrate for glycine‐*N*‐methyltransferase, an enzyme that regulates the pool of *S*‐adenosylmethionine (SAM) in the liver. Because SAM is involved in methyl group transfer during DNA methylation, changes in glycine levels could influence genetic stability [50]. Glycine is also the primary amino acid of collagen, the most abundant protein in humans and a key component of the extracellular matrix, including in joints and vascular walls. Consequently, reduced glycine availability may impair collagen turnover, potentially increasing the risk of vascular complications and osteoarthritis—conditions for which individuals with Type 2 diabetes are at increased risk [51, 52].

Dietary glycine intake in adults is approximately 3 g/day [53]. In individuals with Type 2 diabetes, oral supplementation with 15 g/day glycine for 3 months has been shown to reduce HbA1c levels, decrease the expression of the Tumor necrosis factor receptor 1, and increase plasma levels of interferon gamma [54]. Similarly, glycine supplementation (15 g/day, 3 months) reduced lipid peroxidation and systolic blood pressure in individuals with metabolic syndrome [55]. Studies in healthy individuals have also demonstrated that a single 5‐g dose improves insulin sensitivity during an euglycemic–hyperinsulinemic clamp and during a glucose challenge [56, 57]. Despite these results, the evidence supporting the metabolic benefits of glycine supplementation in managing Type 2 diabetes remains limited due to the small number of studies performed in humans. Consequently, additional clinical trials are needed to establish the effectiveness of glycine supplementation.

### Glutamine: insights into energy regulation, inflammation, and insulin resistance

Although the role of glycine in Type 2 diabetes is primarily associated with oxidative stress and the dorsal vagal complex inhibition of liver glucose output, glutamine—though similarly an amino acid—contributes to insulin resistance through distinct metabolic pathways. Glutamine and glutamate are interconvertible polar amino acids involved in numerous cellular processes, including tricarboxylic acid cycle (TCA) anaplerosis. Circulating levels of glutamine and glutamate exhibit an inverse correlation, with glutamine reduced and glutamate elevated both before and after the onset of Type 2 diabetes [35, 36, 58–61]. Accordingly, plasma glutamine levels and the glutamine‐to‐glutamate ratio are strongly associated with insulin sensitivity [58, 59].

The expression of glutamine‐metabolizing proteins—such as glutamine synthetase (GLUL) and glutaminase (GLS)—is altered in white adipose tissue from individuals with obesity [59, 62] and is normalized after bariatric surgery [62]. Adipose tissue takes up glutamate and releases glutamine [63], and mice with adipose tissue‐specific knockout of *GLS* display altered plasma profiles of both glutamine and glutamate [59]. Conversely, neither *GLUL* nor *GLS* appears to be altered in skeletal muscle [58], which is the primary source of endogenous glutamine. Thus, the metabolism of glutamine in white adipose tissue plays a preponderant role in regulating the plasma glutamine‐to‐glutamate ratio in the context of insulin resistance and obesity. Additionally, glutamine contributes to gluconeogenesis, a process that is enhanced in Type 2 diabetes, further exacerbating the reduction in circulating glutamine levels [64].

Glutamine acts as a potent glucagon‐like peptide 1 (GLP‐1) secretagogue by elevating both cytosolic Ca^2+^ and cAMP levels in intestinal L cells [65]. Furthermore, glutamine is converted into α‐ketoglutarate—a critical intermediate in the TCA cycle—making it an essential substrate for mitochondrial energy production. Consequently, reduced glutaminolysis has been associated with increased glucose utilization in human adipocytes and elevated energy expenditure in both lean and insulin‐resistant mouse models [59]. Decreased glutamine metabolism results in the activation of proinflammatory genes by increasing chromatin *O*‐GlcNAcylation in human adipocytes [62], whereas glutamine availability regulates the balance between T helper 1 cells and regulatory T cells [66]. Therefore, glutamine plays a role in the regulation of immunometabolism, and its reduction may contribute to a state of low‐grade chronic inflammation characteristic of obesity and Type 2 diabetes. In addition to these specific functions, glutamine plays a role in inter‐organ nitrogen exchange via ammonia and serves as a substrate for nucleotide synthesis. Due to the pleiotropic and indirect nature of these effects, establishing clear associations between glutamine availability and its diverse physiological impacts is challenging.

A single dose of glutamine (30 g) is sufficient to increase both fasting and postprandial circulating concentrations of GLP‐1, glucagon, and insulin in both lean and obese individuals, regardless of diabetes status [67, 68]. In contrast, encapsulated glutamine totaling 6 g failed to recapitulate these findings [69], suggesting that a higher therapeutic dose is required to improve glycemia. Indeed, glutamine supplementation (30 g/day for 6 weeks) significantly reduced body fat mass, waist circumference, fasting blood glucose, and HbA1c in individuals with Type 2 diabetes [70], whereas 15 g/day for 4 weeks reduced HbA1c and improved glucose tolerance, although these effects may be due to a mild increase in plasma volume [71]. Although the role of glutamine as a potent effector of GLP‐1 secretion in intestinal L cells is well established, the potential anti‐inflammatory effects of glutamine supplementation remain in a preclinical phase [58, 62] and warrant further investigation.

#### *N*‐Lactoyl‐phenylalanine: a novel link between metabolism and diabetes

Beyond the known amino acids, untargeted metabolomics continues to identify novel biomarkers—such as *N*‐lactoyl‐phenylalanine (Lac‐Phe)—which could alter the landscape of diabetes diagnosis and treatment. *N*‐lactoyl amino acids were identified in human plasma less than a decade ago [72] and were often misidentified as 1‐carboxyethyl amino acids. With advancements in metabolomic technologies, these metabolites are gaining increasing research attention. Among them, Lac‐Phe—a conjugate of lactate and phenylalanine—is the most abundant. Circulating Lac‐Phe has been identified as a risk indicator for diabetes pathogenesis—showing an association with HbA1c progression rates [6]—and an increased risk of diabetic retinopathy in patients with Type 2 diabetes [73]. Systematic reanalysis and correction of previously mislabeled Lac‐Phe in older studies have also demonstrated elevated serum concentrations of this metabolite in Type 2 diabetes [74, 75]. Paradoxically, at supraphysiological doses, Lac‐Phe suppresses food intake, reduces body fat, and improves glucose tolerance in obese mice [76]. This metabolite is also induced by exercise in both mice and humans [76]. Furthermore, higher levels of exercise‐induced Lac‐Phe were associated with greater reductions in abdominal subcutaneous fat in young obese individuals following an 8‐week training protocol [77]. This apparent contradiction was recently addressed by the discovery that Lac‐Phe levels increase in response to both acute and chronic metformin administration [75]. Reanalysis of older studies showed that Lac‐Phe was elevated only in individuals with Type 2 diabetes treated with metformin [75]. As metformin is a first‐line treatment for Type 2 diabetes, this finding explains the frequent observation of elevated Lac‐Phe levels in this patient group.

Exercise and metformin increase Lac‐Phe by enhancing glycolytic flux and lactate production, which is then fused with phenylalanine through the action of the enzyme carnosine dipeptidase 2 [72]. Although the molecular mechanisms explaining its appetite‐suppressing effects remain unexplored, Lac‐Phe has been hypothesized to act as a paracrine or endocrine signaling molecule targeting appetite‐regulating neurons, potentially through binding to a G‐protein‐coupled receptor sensor [78].

Despite its potential, Lac‐Phe remains a promising yet underexplored metabolite, with limited research available at both clinical and preclinical levels. Additionally, Lac‐Phe is not orally bioavailable, and studies in animal models have used high pharmacological levels [76], complicating its evaluation as a therapeutic supplement. Nevertheless, the discovery of Lac‐Phe underscores the transformative potential of advanced metabolomic technologies to identify novel biomarkers and mediators of insulin resistance, even within older datasets.

## Lipids and Type 2 diabetes pathophysiology

Lipids play a central role in the pathogenesis of Type 2 diabetes, as dysregulated lipid metabolism contributes to insulin resistance and metabolic stress (Fig. 2b). Altered lipid handling promotes ectopic fat deposition in non‐adipose tissues, such as the liver and skeletal muscle, which interferes with insulin signaling pathways. Ectopic fat accumulation and defective fatty acid catabolism are associated with lipotoxicity, wherein toxic lipid intermediates disrupt cellular processes and exacerbate insulin resistance. These alterations establish a deleterious feedback loop in which lipid‐induced metabolic stress further aggravates insulin resistance, driving the progression of Type 2 diabetes.

### Sphingolipids: key contributors to insulin resistance

Having established the central role of lipids in insulin resistance and metabolic stress, it is crucial to delve deeper into specific lipid species that have a profound impact on diabetes pathophysiology. One such group is sphingolipids, which play a pivotal role in the development of insulin resistance. Sphingolipids—a class of lipids characterized by a sphingoid base backbone—are divided into sub‐groups, such as glycosphingolipids, sphingomyelins, and ceramides. Among these, ceramides and sphingomyelins are strongly linked to Type 2 diabetes and metabolic syndrome risk [79, 80, 81]. Ceramides are regarded as potent lipotoxic disruptors of metabolism. Long‐chain and very long‐chain ceramides are highly correlated with insulin resistance [82, 83, 84]. Elevated ceramide levels in skeletal muscle [85], liver [86], and adipose tissue [87] have been shown to correlate with reduced insulin sensitivity, strongly implicating aberrant ceramide metabolism in key metabolic tissues. For instance, the expression of the ceramide synthase, *CERS6*, in white adipose tissue is inversely correlated with glucose infusion rates during euglycemic–hyperinsulinemic clamps [88]. Certain sphingolipid species, such as hydroxysphingomyelins and hexosylceramides, are associated with improved glucose homeostasis [81]. Plasma sphingomyelin levels are positively linked with lower HOMA‐IR values, even in individuals with normal body mass index (BMI) [82].

### Ceramide‐mediated insulin resistance: mechanisms and tissue‐specific impacts

Within the class of sphingolipids, ceramides stand out due to their strong association with insulin resistance and metabolic dysfunction. This section explores the mechanisms by which ceramides specifically contribute to these metabolic disruptions. The liver—as a central hub for ceramide metabolism—plays a key role in the connection between ceramides and non‐alcoholic fatty liver disease (NAFLD), a common comorbidity in Type 2 diabetes [89, 90]. Serum ceramide levels are elevated in patients with NAFLD compared to both controls and patients with chronic hepatitis B [91], indicating a specific association between ceramides and fatty liver rather than general liver damage. Moreover, hepatic pro‐ceramide gene expression is increased in non‐alcoholic steatohepatitis. Lifestyle interventions that reduce hepatic lipid content over 1 year also decrease pro‐ceramide gene expression and serum ceramide levels [92].

Ceramides have a direct role in impairing insulin signaling. In myotubes [93] and adipocytes [94], ceramides activate protein kinase C zeta, which inhibits Akt, a central node in the insulin signaling pathway. In hepatocytes, ceramides disrupt the mitochondrial electron transport chain and inhibit fatty acid oxidation, further impairing insulin signaling through reduced Akt activation [95]. Mechanistic evidence distinguishes ceramides from other lipid biomarkers, supporting a causal role in the development of insulin resistance.

Dietary intake of saturated fatty acids drives increased ceramide synthesis and circulating levels compared to unsaturated fatty acids (Table 2), underscoring the importance of limiting saturated fat consumption [96, 97]. Interventions such as bariatric surgery reduce skeletal muscle sphingolipid levels, with exercise further decreasing ceramide content in skeletal muscle [98]. The anti‐diabetic drug pioglitazone is an effective approach to reducing ceramides in insulin‐resistant patients [99]. Although strategies to stimulate ceramide degradation or inhibit ceramide biosynthesis have shown promise in alleviating insulin resistance and related metabolic disorders in rodent models [100], the development of drugs targeting ceramide metabolism has been hindered by toxicity concerns, as observed with fumonisin B1 analogs [101].

**Table 2:** Effects of diet intervention on metabolite levels.

Metabolite	Diet intervention
Branched‐chain amino acids	Meats, fish, and dairy products are major food sources of BCAA, but there is an overall weak association between BCAA intake and circulating levels of BCAA [102, 103] In a prospective study including meat‐eaters, fish‐eaters, vegetarians, and vegans, no major differences in BCAA content were found [104] A 4‐week meat‐rich diet is associated with increased BCAA levels compared to a vegan diet [105] Plasma levels of leucine and valine are lower in individuals following a low‐glycemic index diet compared to low‐fat diet [106] Low protein diet during overfeeding is associated with decreased valine levels [107] A ketogenic diet increases serum BCAA [108]
Phenylalanine	A 7‐day milk‐based high‐protein diet is associated with lower phenylalanine levels [109] In a prospective study including meat‐eaters, fish‐eaters, vegetarians, and vegans, no differences in phenylalanine content were found [104] Dietary protein content during overfeeding is positively associated with increased phenylalanine levels [107]
Glutamine	A 4‐week vegan diet is associated with increased glutamine levels compared to a meat‐rich diet [105] A 7‐day milk‐based high‐protein diet is associated with lower glutamine [109] Low protein diet during overfeeding is associated with increased glutamine levels [107] A ketogenic diet decreases serum glutamine levels [108]
Glycine	A 4‐week vegan diet is associated with increased glycine levels compared to a meat‐rich diet [105] In a prospective study including meat‐eaters, fish‐eaters, vegetarians, and vegans, vegans had the highest concentration of glycine, whereas meat‐eaters had the lowest [104] Plasma levels of glycine are higher in individuals following a low‐glycemic index diet compared to high‐glycemic index diet [106] A 7‐day milk‐based high‐protein diet is associated with lower glycine levels [109] Low‐protein diet during overfeeding is associated with increased glycine levels [107]
Ceramides	Dietary intake of saturated fatty acids drives increased ceramide synthesis and circulating levels compared to unsaturated fatty acids [96, 97]
Acylcarnitines	Ketogenic diets elevate serum acylcarnitines while reducing urinary levels, suggesting increased fatty acid oxidation [108] Butter intake is strongly associated with concentrations of C9:0 and C11:0 acylcarnitines, whereas vegetable oil intake is inversely associated with concentrations of C13:0 and C14:0 acylcarnitines [110]
Phosphatidylcholines	Low‐glycemic diets decrease phosphatidylcholine levels [106] Caloric restriction produces inconsistent outcomes depending on the metabolic context and individual variability [111, 112]
Lysophosphatidylcholines	Low‐glycemic and low‐fat diets increase lysophosphatidylcholine levels [106]
Ketone bodies	Ketogenic diets (high fat, moderate protein, and very low carbohydrate intake) leads to increased production of ketone bodies [108] Very low‐calorie diets promote utilization of fat for energy, resulting in elevated ketone levels [113]
Lactate	A 4‐week diet with low glycemic index and/or reduced carbohydrate content lower plasma lactate concentration [114]
NAD^+^	Dietary NAD^+^ is mainly hydrolyzed in the small intestine [115] Niacin, a key precursor of NAD^+^, is abundant in western diets [116] Energy overload (e.g., high‐fat diet) decreases the NAD/NADH ratio in rodent models [117]

*Note*: This table summarizes how various diets and dietary interventions affect key metabolite levels, highlighting relationships that may influence metabolic processes and cardiometabolic diseases such as obesity or Type 2 diabetes. Circulating amino acid concentrations do not necessarily mirror dietary amino acid intake, suggesting that amino acid homeostasis is tightly regulated. Conversely, factors, such as sex, age, metabolic health, and feeding status, which are not addressed in this table, appear to have a profound impact on the concentration of circulating metabolites.

Abbreviation: NAD^+^, nicotinamide adenine dinucleotide.

### Acylcarnitine dysregulation: implications for insulin resistance and metabolic health

Although ceramides have a direct role in impairing insulin signaling, another important lipid class, acylcarnitines, also plays a central role in metabolic regulation. Acylcarnitines are key intermediates in fatty acid oxidation, and their altered metabolism has been implicated in insulin resistance. Acylcarnitines act as intermediates that facilitate the transport of acyl groups from the cytosol into mitochondria, where they undergo fatty acid oxidation. Although medium‐chain fatty acids can diffuse freely across the mitochondrial membrane, long‐chain fatty acids require esterification to l‐carnitine for transport to mitochondria via the carnitine shuttle. This process leads to the formation of acylcarnitines. The quantification of acylcarnitines serves as a proxy for assessing fatty acid oxidation, with elevated levels often indicating impaired beta oxidation. Notably, the plasma acylcarnitine profile is considered a strong biomarker and risk factor for Type 2 diabetes [8, 118–120]. For example, short‐ and medium‐chain acylcarnitines are elevated in the liver, plasma, and pancreatic islets of individuals with Type 2 diabetes [121]. Even in men without diabetes, medium‐chain acylcarnitines are inversely associated with insulin sensitivity [122]. Very short‐chain acylcarnitines are reduced in skeletal muscle of individuals with Type 2 diabetes, underscoring the tissue‐specific variations in acylcarnitine metabolism during metabolic dysfunction [121]. The diversity of acylcarnitine species and the complexity of quantification methods often result in inconsistencies across studies. For example, decreased levels of long‐chain C18 acylcarnitine have been reported as predictors of Type 2 diabetes [123]. Similarly, while increased short‐chain acylcarnitines and decreased medium‐ and long‐chain acylcarnitines are observed in some individuals with Type 2 diabetes [124], other studies report contrary findings. Elevated acylcarnitine levels are primarily attributed to disruptions in mitochondrial fatty acid oxidation and BCAA catabolism, resulting in the accumulation of intermediate acylcarnitines in the blood [125]. C3 and C5 acylcarnitines, intermediates of BCAA metabolism, may contribute to this accumulation when BCAA catabolism is impaired.

Acylcarnitines likely exacerbate insulin resistance through multiple mechanisms. They increase the intracellular abundance of fatty acyl CoAs and diacylglycerol, which inhibit insulin signaling [126, 127]. Additionally, they contribute to ceramide synthesis, which directly impairs insulin signaling via Akt [93, 94]. Acylcarnitine‐driven fatty acid oxidation may also produce excessive acetyl CoA, creating feedback inhibition that dampens glycolysis and glucose uptake [128]. Acylcarnitines are not only effective biomarkers but also represent potential therapeutic targets for insulin resistance and related disorders. Strategies to modulate acylcarnitine levels focus on enhancing fatty acid oxidation. For example, exercise transiently increases circulating acylcarnitines while reducing urinary levels, reflecting the utilization of acylcarnitines as an energy source [129]. Similarly, ketogenic diets in healthy individuals elevate serum acylcarnitines while reducing urinary levels (Table 2), suggesting increased fatty acid oxidation [108].

### Phospholipid metabolism, dietary interventions, and impact on insulin sensitivity

Beyond ceramides and acylcarnitines, phospholipids such as phosphatidylcholine (PC) and lysophosphatidylcholine (LPC) have also emerged as important players in metabolic dysfunction. Their roles in lipid metabolism and insulin sensitivity warrant further examination. PC is the most abundant phospholipid, comprising 40%–50% of cellular phospholipid content. Numerous studies have reported reduced circulating PC levels in individuals with Type 2 diabetes [130, 131, 132, 133], though some studies indicate a positive correlation between certain PCs and BMI [134, 135]. The PC family exhibits structural heterogeneity, which likely accounts for conflicting findings. PCs with one ester‐ and one ether‐linked fatty acid are inversely associated with BMI and obesity [130, 132, 135, 136], whereas PCs with two ester‐linked fatty acids show a positive correlation [132, 134, 136]. This divergence can be attributed to distinct metabolic roles. Ester‐linked PCs primarily support very low‐density lipoprotein (VLDL) release [137], a process that is upregulated in states of increased fatty acid availability, such as obesity. In contrast, ether‐linked PCs act as antioxidants, mitigating lipotoxicity [138]. Elevated fatty acid availability in obesity drives greater VLDL synthesis, which may increase ester‐linked PC levels, whereas the heightened lipotoxic stress depletes antioxidant‐linked PCs.

LPCs, produced through cleavage of PCs, play important roles in mitochondrial membranes and oxidized low‐density lipoproteins. Skeletal muscle biopsies from insulin‐resistant individuals show reduced LPC levels alongside elevated PCs, suggesting opposing roles in metabolic dysfunction [133]. Plasma LPCs negatively correlate with BMI [131, 132, 139, 140] and are reduced in Type 2 diabetes [133, 136, 141], with the exception of lysoalkylphosphatidylcholine, which shows a positive association [132, 135, 136]. Structural differences, such as the substitution of an acyl chain with an alkyl chain, may alter signaling pathways via G‐protein‐coupled receptors. This structural change may affect signaling pathways typically mediated by circulating LPCs, including pathways involved in glucose‐driven insulin secretion by pancreatic β cells [142, 143, 144]. Similarly, reduced circulating levels of LPCs may have an attenuated action as secretagogues for insulin release.

Dietary interventions produce varied effects on PC and LPC levels, reflecting the complexity of lipid metabolism (Table 2). Low‐glycemic and low‐fat diets have been shown to increase LPC levels while simultaneously reducing PC levels [106]. Caloric restriction, however, produces inconsistent outcomes, with some studies reporting increases in PC levels [111] and others noting decreases, depending on the metabolic context and individual variability [112]. Experimental evidence from rodent studies suggests that PC supplementation exerts anti‐glycemic effects [145], though similar evidence in humans remains limited due to a lack of clinical trials. Observational studies further complicate the picture, indicating PC consumption may elevate the risk for Type 2 diabetes [146], possibly due to the production of trimethylamine, a gut‐derived metabolite strongly linked to Type 2 diabetes [147, 148]. Thus, although PCs and LPCs are robust biomarkers for metabolic dysfunction, understanding the precise role and therapeutic potential of these metabolites requires further investigation, particularly in the context of dietary interventions and human clinical outcomes.

## Energetic stress metabolites

Energetic stress refers to a condition in which cellular energy demands exceed the available supply, triggering compensatory metabolic pathways to restore energy balance. Energy stress metabolites are central to this adaptive response, playing crucial roles in maintaining metabolic equilibrium and responding to shifts in energy availability. These metabolites regulate key processes essential for preserving insulin sensitivity and overall metabolic health. Variations in the concentrations and activities of these metabolites often reflect disruptions in energy homeostasis, such as those observed in obesity and Type 2 diabetes (Fig. 2c). Beyond serving as markers of metabolic imbalance, energy stress metabolites act as signaling molecules, activating adaptive pathways that enhance metabolic flexibility. This adaptive capacity may mitigate insulin resistance and improve metabolic function. However, due to the ubiquitous roles of these metabolites in cellular biology, establishing a direct causal relationship between specific metabolites and the onset of diabetes remains challenging.

### Ketone bodies: metabolic adaptors in energy deficiency and insulin sensitivity

Among the energetic stress metabolites, ketone bodies—particularly β‐hydroxybutyrate—have gained attention for their potential role in improving insulin sensitivity. Their therapeutic applications—especially in ketogenic diets—are explored in the following section. Ketone bodies are the products of liver ketogenesis, a process triggered by conditions of low insulin and glucose availability, such as fasting, starvation, intense exercise, or untreated Type 1 diabetes. During ketogenesis, oxaloacetate exits the TCA cycle prematurely to support gluconeogenesis, leading to the accumulation of acetyl CoA. This excessive acetyl CoA is converted into the ketone bodies acetoacetate and β‐hydroxybutyrate, with acetoacetate also capable of being further metabolized into acetone. This section explores the mechanisms by which ketone bodies—particularly β‐hydroxybutyrate—improve insulin sensitivity and glucose tolerance, with potential applications for therapeutic strategies.

Ketogenic diets, characterized by a high fat intake (>60% of total calories) and minimal carbohydrate consumption (<5%–10%), have shown beneficial effects for individuals with Type 2 diabetes. Meta‐analyses indicate improvements in key outcomes such as body weight, HbA1c, and fasting blood glucose levels [149]. The benefits of ketogenic diets for weight loss and glycemic control in individuals with obesity and Type 2 diabetes often surpass those achieved with low‐fat or low‐carbohydrate diets, even without caloric restriction [113, 150–152]. However, low long‐term adherence to ketogenic diets may limit effectiveness in improving glycemic control when compared to a chronic intervention. Although HbA1c reduction is apparent within the first 6 months of a ketogenic diet, the effect diminishes after 2 years [153], suggesting the diet's utility as a short‐term strategy rather than a chronic solution for managing glycemic control.

The primary mechanism by which ketogenic diets enhance insulin sensitivity is through a reduction in circulating glucose. Additionally, β‐hydroxybutyrate acts as a signaling molecule by binding to the GPR109a receptor [154], a pathway linked to improved insulin sensitivity in humans, albeit via another agonist [155], and improved glucose tolerance in preclinical models. Binding of β‐hydroxybutyrate to GPR109a activates signaling cascades involving protein kinase A and peroxisome proliferator‐activated receptor γ [156], which enhance insulin sensitivity. Activation of hepatic AMP‐activated protein kinase by β‐hydroxybutyrate further contributes to improved insulin sensitivity [157].

In addition to ketogenic diets, sodium‐glucose cotransporter 2 (SGLT‐2) inhibitors also elevate circulating levels of β‐hydroxybutyrate. These inhibitors primarily exert therapeutic effects by reducing renal glucose reabsorption but are associated with cardioprotective effects in Type 2 diabetes. The benefit is thought to arise from enhanced fatty acid oxidation in cardiac cells [158]. SGLT‐2 inhibition—alongside increased β‐hydroxybutyrate levels—has been linked to reduced liver fat content, strongly suggesting a role in enhancing hepatic fatty acid oxidation [159]. These findings highlight β‐hydroxybutyrate as a potential therapeutic mediator, offering metabolic and cardioprotective benefits through various treatment modalities beyond ketogenic diets.

### Lactate: from byproduct to key regulator of metabolism and insulin sensitivity

In addition to ketone bodies, lactate has emerged as an important regulator of metabolism. Although traditionally viewed as a byproduct of anaerobic metabolism, lactate has emerged as a key metabolic intermediate involved in tissue‐to‐tissue energy exchange, regulation of redox balance, and signaling in various biological processes [160]. This section delves into the mechanisms by which lactate affects glucose utilization and its contribution to the metabolic dysfunction seen in Type 2 diabetes. Lactate's role in insulin secretion and lipolysis inhibition through receptor activation is also highlighted, suggesting its complex effects on metabolic health. Elevated circulating lactate levels are considered a risk factor for Type 2 diabetes [161, 162, 163, 164], potentially signaling early impairments in oxidative metabolism. Individuals with Type 2 diabetes who carry a polymorphism in the lactate transporter gene *MCT1*—associated with enhanced lactate transport—display lower fasting plasma glucose compared to those with the wild‐type gene [165]. This suggests that enhanced lactate clearance capacity may contribute to better glucose control.

In insulin‐resistant skeletal muscle, glycolysis and lactate production are markedly increased [166]. Concomitantly, expression of the lactate transporter MCT1 is reduced in the skeletal muscle of individuals with Type 2 diabetes [166]. MCT1 facilitates the transport of lactate from glycolytic fibers to oxidative fibers, where it is oxidized to pyruvate and enters the TCA cycle [167]. Reduced MCT1 expression not only deprives oxidative muscle cells of a key fuel substrate but also contributes to the elevated circulating lactate levels. Lactate production is further elevated during hyperinsulinemic–euglycemic clamp studies, a condition that mimics the hyperinsulinemia characteristic of early stages of Type 2 diabetes [168].

Whether elevated lactate directly contributes to insulin resistance remains unclear, but evidence suggests it may play a role. In the liver, lactate is converted to glucose, and excessive blood lactate could exacerbate hyperglycemia in the context of hepatic insulin resistance. Furthermore, “lactate clamp” studies demonstrate that lactate reduces glucose oxidation [169], and ex vivo experiments indicate that lactate inhibits glycolytic enzyme activity [170]. These observations suggest a feedback loop in which elevated lactate reduces glucose utilization and oxidation, potentially exacerbating metabolic dysfunction.

Lactate stimulates insulin release in β‐cells when combined with other insulin secretagogues, such as ketone bodies [171]. Although this may enhance insulin‐mediated responses under physiological conditions—such as during exercise—chronic hyperlactatemia may worsen hyperinsulinemia and β‐cell exhaustion in Type 2 diabetes. Lactate additionally acts as a signaling molecule to inhibit lipolysis via activation of the G‐protein‐coupled receptor hydroxycarboxylic acid receptor 1 [172]. However, in vitro studies suggest chronic lactate exposure can either impair or improve mitochondrial respiration and may enhance reactive oxygen species production, depending on experimental conditions [173, 174].

Beyond metabolic regulation, lactate has been shown to modify proteins post‐translationally through a process termed lactylation, where lactyl groups are covalently attached to lysine residues [175]. Histone lactylation activates homeostatic gene expression in macrophages [175], positioning lactate as a link between metabolism and immunity. Consistent with higher lactate in prediabetic conditions, protein lactylation is elevated in skeletal muscle from obese, insulin‐resistant women compared to lean controls [176]. Despite these findings, the potential role of lactate in Type 2 diabetes pathophysiology remains speculative. Most studies are preliminary and have yet to be replicated, whereas changes in circulating lactate often coincide with alterations in whole‐body glucose metabolism, local pH, and redox status.

### NAD^+^ and related metabolites: central players in energy homeostasis and insulin sensitivity

Nicotinamide adenine dinucleotide (NAD⁺) is an essential redox cofactor involved in cellular energy metabolism, facilitating electron transfer from glycolysis and the TCA cycle to the electron transport chain for ATP production. Beyond its primary role, NAD⁺ also serves as a substrate for enzymes such as deacylase and poly(ADP‐ribose) polymerase, which are involved in cellular signaling and DNA repair. The ratio between NAD^+^ and its reduced form NADH (NAD⁺/NADH ratio) is a key indicator of metabolic homeostasis. A high NAD⁺/NADH ratio reflects active oxidative phosphorylation and is commonly associated with favorable metabolic health. This section discusses the role of NAD⁺ in energy metabolism, its association with Type 2 diabetes, and how NAD⁺‐dependent enzymes—such as sirtuins—impact insulin sensitivity and metabolic function.

Although direct evidence linking NAD⁺ to metabolic diseases remains limited, a cross‐sectional study has found a positive association between whole‐blood NAD⁺ levels and the prevalence of metabolic disease [177]. The enzyme nicotinamide *N*‐methyltransferase (*NNMT*) impedes NAD⁺ synthesis by converting nicotinamide into methylated nicotinamide. A single‐nucleotide polymorphism in the *NNMT* gene is associated with Type 2 diabetes [178]. Elevated plasma levels of methylated nicotinamide and increased *NNMT* expression in white adipose tissue have been observed in individuals with Type 2 diabetes, with plasma methylated nicotinamide correlating positively with fasting glucose [179, 180]. These findings suggest that increased *NNMT* activity may lower NAD⁺ levels in white adipose tissue during obesity, as shown by reductions in *NNMT* expression and concurrent increases in NAD⁺ levels in adipose tissue following bariatric surgery in obese individuals [179, 181]. These observations may indicate a specific role for NAD^+^ in white adipose tissue metabolic dysfunction. This is further supported by the downregulation of NAD^+^‐producing genes in adipose tissue of the heavier co‐twin in BMI‐discordant twin studies [182].

The metabolic effects of altered NAD⁺ levels are likely mediated, at least in part, by NAD⁺‐dependent Sirtuin enzymes (SIRT1‐7). The loss of SIRT1 specifically in adipose tissue impairs insulin sensitivity [183], whereas SIRT1 deletion in the liver results in hepatic steatosis [184]. SIRT1 expression in skeletal muscle and adipose tissue is frequently reduced in individuals with obesity and Type 2 diabetes [185, 186], and this reduction is associated with insulin resistance [186].

### NAD⁺ precursor supplements: potential benefits in metabolic disease treatment

Given the apparent role of NAD⁺ in metabolic health, supplementation with NAD⁺ precursors such as nicotinic acid and nicotinamide riboside (NR) has been explored as a potential strategy for managing metabolic diseases, including Type 2 diabetes. This section reviews the evidence on the efficacy of NAD⁺ precursors in improving insulin sensitivity, glucose regulation, and dyslipidemia. Although the results are mixed, there is promise in certain contexts, particularly in combination with exercise or for managing lipid metabolism. NAD⁺ precursors effectively increase NAD⁺ levels in human white blood cells and skeletal muscle [187, 188]. Nicotinic acid, an NAD^+^ precursor, has been used for decades as an antilipolytic agent [189], whereas its analog acipimox is currently being investigated as a treatment for metabolic disorders. In healthy individuals, acipimox has been shown to reduce fasting free fatty acids without affecting insulin or glucose levels [190]. In individuals with Type 2 diabetes, the effects are more variable. Some studies have demonstrated reductions in fasting insulin, glucose, and mean glucose during oral glucose tolerance tests [191], but its effects on improving insulin sensitivity—as measured by a euglycemic clamp—have been inconsistent in other studies [192]. However, acipimox may be most effective when used in conjunction with exercise, as it significantly lowers post‐exercise insulin and glucose levels in individuals with Type 2 diabetes [193]. NR—another NAD⁺ precursor—has been explored as a treatment for metabolic disorders but has generally yielded minimal results. Studies in obese and insulin‐resistant individuals have shown that NR supplementation does not significantly affect metabolic indicators, such as glucose, insulin, GLP‐1, or body composition measures, nor does it appear to improve insulin sensitivity or lipid metabolism [103, 104]. Overall, although NAD⁺ precursors may offer limited value in treating insulin resistance or glucose regulation independently, they show promise in specific contexts, such as in combination with exercise, and have an established role in managing dyslipidemia.

## Conclusions

The field of metabolomics has advanced our understanding of cardiometabolic diseases, particularly in identifying key metabolites associated with insulin resistance and diabetes. Metabolites, such as BCAAs, ceramides, glycine, and glutamine, have emerged as promising biomarkers for early diagnosis, risk assessment, and targeted therapeutic interventions. Elevated levels of BCAAs and ceramides, for instance, are strongly linked to insulin resistance, whereas glycine shows potential as a therapeutic target with its inverse correlation to insulin resistance. Energy stress metabolites such as ketone bodies, lactate, and NAD⁺ also play critical roles in regulating metabolic health and insulin sensitivity, offering potential avenues for therapeutic development. Ketogenic diets—which boost ketone body production—and NAD⁺ precursor supplementation have shown promise in improving insulin sensitivity, particularly when combined with exercise, although long‐term efficacy remains an area of ongoing research. The novel biomarker Lac‐Phe further highlights the complexity of metabolic regulation and its potential for therapeutic application. Ultimately, these metabolites form intricate metabolic networks, creating a complex landscape of biochemical interactions (Fig. 2d). Understanding the precise roles of these metabolites and their impact on metabolic pathways could lead to personalized treatment plans, offering more effective management of diabetes and related conditions. Clinically, metabolite‐based diagnostics and precision medicine approaches could potentially aid in the prevention, early detection, and tailored therapies for individuals at risk of or living with cardiometabolic diseases.

## Author contributions

**David Rizo‐Roca**: Conceptualization; writing—original draft; writing—review and editing; created figures. **John D. Henderson**: Writing—original draft. **Juleen R. Zierath**: Conceptualization; writing—original draft; writing—review and editing; supervision.

## Conflict of interest statement

The authors declare no conflicts of interest.

## Funding information

J.R.Z. is supported by the Swedish Research Council (2015‐00165), the Novo Nordisk Foundation (NNF22OC0077741, NNF17OC0030088 and NNF23SA0084103), a Wallenberg Scholars Award from the Knut and Alice Wallenberg Foundation (KAW 2023.0312), and the European Research Council (ERC‐2023‐AdG 101142093). The Novo Nordisk Foundation Center for Basic Metabolic Research (CBMR) is an independent research center at the Faculty of Health and Medical Sciences, University of Copenhagen, Denmark, partially funded by an unrestricted donation from the Novo Nordisk Foundation (NNF18CC0034900, NNF23SA0084103).

## Data Availability

Data sharing is not applicable to this article as no new data were created or analyzed in this study.
